# Prevalence and Predictors of Depressive Symptom Trajectories in Older Adults: A Systematic Review

**DOI:** 10.1002/brb3.71793

**Published:** 2026-10-06

**Authors:** Yuying Li, Qiuyan Liu, Xue Dong

**Affiliations:** ^1^ Department of Nursing Changchun University of Chinese Medicine Changchun Jilin China

**Keywords:** geriatric depression, risk factors, systematic review, trajectory analysis

## Abstract

**Purpose:**

To describe the distribution patterns and cross‐study heterogeneity of depressive symptom trajectories in older adults and to identify consistent predictors of high‐symptom trajectories for targeted intervention strategies.

**Methods:**

Following PRISMA 2020 guidelines, six databases were searched from April 2010 to January 2026 for longitudinal studies using latent variable mixture models in adults aged ≥ 60 years. Study quality was assessed using the Newcastle–Ottawa Scale. Descriptive synthesis was employed due to methodological heterogeneity.

**Results:**

Twenty‐one studies (median *N* = 3428; 26 observations after accounting for within‐study stratification by sex, poverty status, and country) met inclusion criteria. High‐symptom trajectories were identified in 18 of 21 studies (0%–42.2%). Low‐symptom trajectories predominated (12.4%–84.5%). Consistent risk factors included female sex, functional limitations, and multimorbidity. Higher educational attainment was the most consistent protective factor.

**Conclusions:**

Older adults exhibit heterogeneous depressive symptom trajectories, with clinically significant high‐symptom subgroups universally present but context‐dependent. Modifiable predictors provide evidence for precision prevention strategies. Because estimates are descriptive and derived from heterogeneous instruments, they should be interpreted as heuristic indicative ranges rather than precise global benchmarks. Standardized assessment protocols are needed to enable quantitative synthesis.

## Graphical abstract

This systematic review synthesized 21 longitudinal studies using latent variable mixture models to delineate depressive symptom trajectories in older adults. Three distinct trajectory categories were identified: low‐symptom stable trajectories predominated across settings (12.4%–84.5%), moderate‐symptom trajectories exhibited substantial cross‐study heterogeneity, and clinically significant high‐symptom trajectories ranged from 0% to 42.2% (median 14.3%, IQR 8.6%–33.6%). Consistent risk factors for adverse trajectories included female sex (OR 1.43–3.98), functional limitations, and multimorbidity; higher educational attainment emerged as the most robust protective factor (OR 0.26–0.95). The findings underscore the need for standardized assessment protocols to enable future quantitative meta‐analyses and support the development of targeted precision prevention strategies for high‐risk older adults.

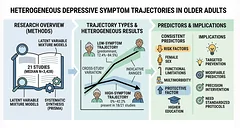

**Abbreviations**
ADL: activities of daily living
CES‐D: Center for Epidemiologic Studies Depression Scale
GBTM: Group‐Based Trajectory Model
GMM: Growth Mixture Model
IADL: instrumental activities of daily living
LCGA: Latent Class Growth Analysis
LCMM: Latent Class Mixed Model
NOS: Newcastle–Ottawa Scale
OR: odds ratio
RRR: relative risk ratio
SD: standard deviation

## 1 Introduction

Geriatric depression has exerted severe negative impacts on the quality of life and long‐term health outcomes of older adults amidst globally accelerating population aging (Gambaro et al. 2022). The disorder is characterized by high prevalence and substantial disability, and is frequently associated with functional decline, cognitive impairment, premature suicide‐related deaths, increased mortality, diminished quality of life, and early nursing home admission (Diniz et al. 2013; van Poelgeest et al. 2021).

Most studies show that geriatric depression symptoms vary significantly across individuals and change dynamically over time (Zheng and Jia 2022; Yuan et al. 2023). Traditional variable‐centered techniques view samples as homogeneous entities, making it difficult to identify this heterogeneity. In contrast, individual‐centered latent variable mixture models assume populations consist of several unobservable subgroups. These models can identify latent trajectory categories, such as persistently low symptoms, persistently high symptoms, late‐onset worsening, and improvement, based on similar change patterns in longitudinal data (Nylund et al. 2007; D. Wang et al. 2025). They further examine the predictors and subsequent health outcomes associated with each category.

However, because of differences in study populations, assessment techniques, and follow‐up designs, current estimates of the quantity, patterns, and influencing factors of trajectory categories vary considerably. Although existing reviews have examined the heterogeneity of depressive symptom trajectories across global populations of various ethnicities, most included studies encompassed all age groups and did not specifically focus on older adults (Musliner et al. 2016). With the intensifying global aging trend, the unique trajectory patterns and predictive factors of geriatric depression require further integration.

Therefore, regarding trajectory characteristics, we propose the following hypotheses: (a) Multiple distinct depressive symptom trajectories will be identified among older adults; (b) although low‐stability trajectories will predominate, clinically significant high‐symptom trajectories will affect a substantial minority; (c) prevalence rates of high‐symptom trajectories will show considerable variation across studies due to demographic characteristics and methodological differences; and (d) members of high‐symptom trajectories will exhibit characteristics such as physical functional decline and low social engagement.

Thus, the primary objective of this review is to describe the distribution patterns and cross‐study heterogeneity of depressive symptom trajectories in older adults, with a focus on high‐symptom subgroups with preventive and therapeutic significance. A secondary objective is to identify consistent predictors of high‐symptom trajectories, providing evidence for developing targeted intervention strategies.

## 2 Materials and Methods

This study was guided by the PRISMA 2020 statement (Page et al. 2021) and was registered in the International Prospective Register of Systematic Reviews (Registration number: CRD420261280625).

### 2.1 Data Sources and Search Strategy

Two researchers searched for papers published between April 2010, when group‐based trajectory modeling became more widely applied in geriatric psychiatry (Nagin and Odgers 2010), and January 2026. Searches were conducted using China National Knowledge Infrastructure (CNKI), Wanfang Data Knowledge Service Platform, China Science and Technology Journal Database (VIP), PubMed, Embase, and Web of Science, using the search (“depress” OR “depressive symptom” OR “depressive disorder”) AND (“older adult” OR “elder” OR “geriatric” OR “late life” OR “aging” OR “ageing”) AND (“trajector” OR “longitudinal course” OR “growth mixture model” OR “latent class” OR “latent profile” OR “group‐based trajectory model” OR “heterogene” OR “subgroup” OR “developmental pattern”). Abstracts were screened, and papers were selected according to the inclusion and exclusion criteria for full‐text screening.

### 2.2 Eligibility Criteria and Article Selection

The criteria for inclusion are the following: (1) observational longitudinal studies (prospective/retrospective cohort or panel surveys) with at least three repeated measures of depressive symptoms (including baseline); (2) study population consisting of community‐dwelling or institutionalized older adults, defined as mean baseline age ≥ 60 years or all participants aged ≥ 60 at enrollment; (3) studies focusing on depressive symptoms measured via validated scales or clinically diagnosed depressive disorders; (4) studies using latent variable mixture models (e.g., Latent Class Growth Analysis [LCGA], Growth Mixture Models [GMM], group‐based trajectory modeling) to identify heterogeneous trajectories; and (5) studies reporting at least two trajectory classes and their parameters, along with associations between baseline predictors (e.g., demographic, clinical, psychosocial factors) and trajectory membership.

The exclusion criteria were as follows: (1) non‐observational designs (e.g., RCTs, cross‐sectional studies, reviews, and conference abstracts); (2) studies focusing on clinical samples with specific physical/psychiatric diseases where depression is not the primary focus; (3) studies with mean baseline age < 60 years were excluded to ensure strict adherence to the geriatric focus of this review. For multinational studies reporting country‐specific subsamples, subsamples with mean baseline age < 60 years were excluded during data extraction, even if the overall study met the age criterion; (4) use of only traditional group‐level statistical methods without trajectory modeling; (5) studies that do not examine predictors of trajectory membership; (6) unavailability of full text; and (7) non‐Chinese/English publications.

### 2.3 Data Extraction and Quality Assessment

Two reviewers independently conducted the systematic literature searches and extracted data in accordance with predefined criteria. Any discrepancies were resolved through consultation with a third reviewer. The following data were extracted from each included study: first author and publication year; country; study design; baseline sample size and final analytical sample; data source; study setting (community/institution); age and gender distribution; key population characteristics; number of follow‐ups and total follow‐up duration; depression assessment method; trajectory analysis model; number and types of trajectories identified (with proportions); and predictor variables (including exposure factors, target trajectories or health outcomes, effect measures, effect sizes, confidence intervals, *p*‐values, adjusted covariates, and nature of evidence). Two independent reviewers assessed study quality using the Newcastle–Ottawa Scale (NOS), with total scores categorized as low (0–3), moderate (4–6), or high (7–9) quality (Stang 2010).

### 2.4 Data Synthesis and Standardization Procedure

To facilitate cross‐study comparison, this study standardized trajectory estimates across included studies by recalibrating depression symptom scores onto the metric of the Center for Epidemiologic Studies Depression Scale (CES‐D) 20‐item version (range 0–60) (Radloff 1977). Specifically, we applied proportional rescaling whereby observed scores were divided by the maximum possible score of the original instrument and multiplied by 60 (e.g., a score of 7 on the EURO‐D [range 0–12] was rescaled as [7/12] × 60 = 35). Symptom cutoff criteria were designated as “Low” (scores 0–< 16), “Moderate” (scores 16–< 24), and “High” (scores 24–60), reflecting the established screening threshold (≥ 16) originally validated by Radloff (Radloff 1977) and the severe symptom threshold (≥ 24) widely applied in geriatric epidemiology (Ruiz‐Grosso et al. 2012; Hertzog et al. 1990).

Regarding trajectory trends, this study categorized them uniformly into ascending, descending, and stable types. (1) Ascending type: statistically significant positive slope; (2) declining type: statistically significant negative slope; (3) stable type: nonsignificant slope with fluctuations around a constant baseline.

Despite heterogeneity in assessment instruments (5‐item to 20‐item versions), visual inspection confirmed that trajectory groups labeled as “high” in original studies generally fell above the rescaled threshold of 24 CES‐D equivalent points, supporting face validity of the standardization approach.

### 2.5 Statistical Analysis

Given substantial between‐study heterogeneity in measurement tools and statistical models, we employed descriptive synthesis rather than meta‐analysis. We calculated median prevalence and interquartile ranges for each trajectory category across studies, specifically examining low‐symptom, moderate‐symptom, high‐symptom, increasing, and decreasing trajectories. To explore potential sources of variation without implying statistical precision, we stratified these descriptive summaries by assessment tool type, specifically ultra‐short scales with 10 or fewer items, standard scales with 11–20 items, and extended scales with more than 20 items, as well as by analytical model, distinguishing between different analytical methods.

To assess the influence of individual studies on observed patterns, we conducted descriptive sensitivity analyses: (a) excluding the largest study to examine small‐study effects; (b) stratifying by publication year (pre‐2020 vs. 2020–2025); and (c) comparing community‐based versus institutional settings. Findings were considered robust if median prevalence estimates remained within similar ranges across these stratifications; (d) restricting to one estimate per overlapping cohort to address nonindependence, retaining the study with the longest follow‐up or largest analytical sample whenever multiple publications drew from the same database (e.g., CHARLS, ELSA, or HRS).

For predictor variables, we employed vote‐counting to tally the frequency of studies reporting significant associations in multivariate models. Predictors were then categorized based on consistency of evidence, with consistent risk factors defined as those significant in three or more studies, inconsistent risk factors as those significant in only one or two studies or showing conflicting results across studies, and consistent protective factors as those showing significant inverse associations in two or more studies. Effect sizes were summarized descriptively as ranges of odds ratios or relative risk ratios rather than pooled estimates.

## 3 Results

A total of 2366 articles were found using systematic retrieval (PubMed: 792 articles, Embase: 118 articles, Web of Science: 1322 articles, CNKI: 57 articles, Wanfang: 28 articles, and VIP: 49 articles). After deleting 312 duplicate records, the remaining 2054 articles were subjected to title and abstract screening, resulting in the elimination of 1947 articles that did not match the theme and study design requirements. Following a full‐text review of the remaining 107 papers, 21 studies (Bao et al. 2023; Borza et al. 2022; Cermakova and Csajbók 2023; de la Torre‐Luque et al. 2019; Galatzer‐Levy and Bonanno 2014; Holmes et al. 2018; Iob et al. 2023; Kuchibhatla et al. 2012; Kumar et al. 2021; H. Lee, Park, et al. 2017; Liang 2020; Liu et al. 2025; Montagnier et al. 2014; Pan and Cao 2023; Ran et al. 2024; Tsang et al. 2025; J. Y. Wang et al. 2023; Xie et al. 2023; Yazawa et al. 2025; Yu et al. 2025; Zhou et al. 2025) were included in the analysis based on predetermined inclusion and exclusion criteria. Figure 1 depicts the complete systematic search and screening method, while Table 1 summarizes the basic characteristics of the included studies.

**FIGURE 1:**
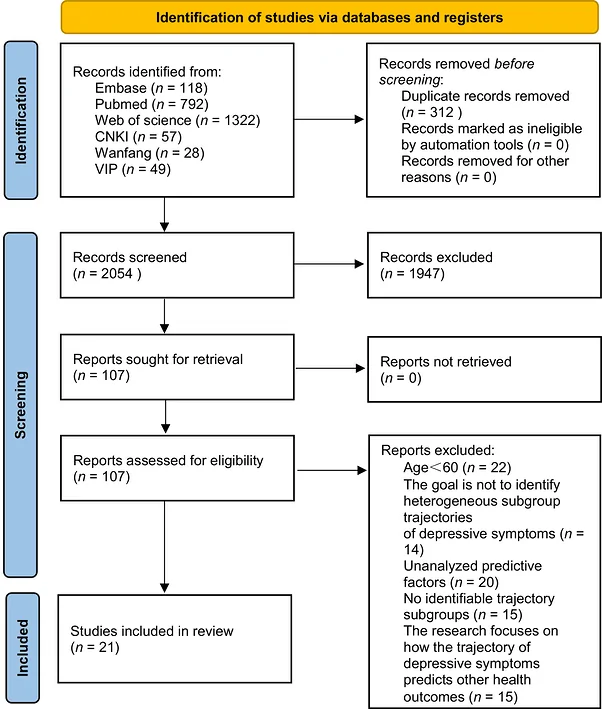
PRISMA 2020 flow diagram of the systematic search and selection process. A total of 2366 records were identified across six databases (PubMed, Embase, Web of Science, CNKI, Wanfang, and VIP). After removing 312 duplicates and screening titles/abstracts, 107 full‐text reports were assessed for eligibility. Note that 21 studies were ultimately included. CNKI, China National Knowledge Infrastructure; VIP, China Science and Technology Journal Database.

**TABLE 1:** Basic characteristics and quality evaluation of the included studies.

Author/publish year	Country	Study design	Participants		Data source	Environment	Age (mean ± SD)	Gender	Quality assessment by NOS (score)
			Original N	Analytic N					
Bao et al. (2023)	China	Retrospective cohort study	5644	2177	CHARLS	Community	76.28 ± 4.67	Female 45.5% Male 54.5%	8
Borza et al. (2022)	Norway	Prospective cohort study	696	656	47 nursing homes in Norway	Institution	84.5 ± 7.5	Female 64.1% Male 35.9%	8
Cermakova and Csajbók (2023)	27 European countries (including Israel)	Retrospective cohort study	140 129	47 010	SHARE	Community	63.0 ± 8.6	Female 56 % Male 44%	7
de la Torre‐Luque et al. (2019)	UK	Retrospective cohort study	13,615	13,203	ELSA	Community	65.07 ± 10.0	Female 52.75% Male 47.25%	8
Galatzer‐Levy and Bonanno (2014)	USA	Retrospective cohort study	2147	2147	HRS	Community	71.1 ± 10.5	Female 51.4% Male 48.6%	7
Holmes et al. (2018)	Australia	Prospective cohort study	423	423	AIBL	Community	69.4 ± 6.6	Female 54.6% Male 45.4%	6
Iob et al. (2023)	UK	Prospective cohort study	3428	3428	ELSA	Community	70.0^a^	Female 55% Male 45%	8
Kuchibhatla et al. (2012)	USA	Prospective cohort study	4162	3973	Duke EPESE	Community	73.3^a^	Female 65% Male 35%	8
Kumar et al. (2021)	USA	Prospective cohort study	5546	3541	ACT	Community	Male 72.9 ± 5.5 Female: 73.8 ± 6.0	Female 59% Male 41%	8
H. Lee, Park, et al. (2017)	South Korea	Prospective cohort study	4162	4129	KLoSA	Community	73.2 ± 6.0	Female 55 % Male 45 %	8
Liang (2020)	China	Prospective cohort study	3522	1984	CHARLS	Community	66.99 ± 5.87	Female 49.7% Male 50.3%	8
Liu et al. (2025)	China	Prospective cohort study	17,705	1832	CHARLS	Community	/	Female 45.5% Male 54.5%	8
Montagnier et al. (2014)	France	Prospective cohort study	2590	2590	PAQUID Study	Community	Male 73.7 ± 0.16 Female 74.8 ± 0.19	Female 59.1% Male 40.9%	7
Pan and Cao (2023)	China	Retrospective cohort study	17,708	5734	CHARLS	Community	68.36^a^	Female 49.9% Male 50.1%	8
Ran et al. (2024)	USA, UK, Mexico	prospective cohort study	19,479	19,479	HRS, ELSA, MHAS	Community	USA: 65.3 ± 9.2 UK: 65.5 ± 8.3 Mexico: 62.4 ± 7.8	USA: Female 57.6%, Male 42.4% UK: Female 53.7%, Male 46.3% Mexico: Female 53.4%, Male 46.6%	7
Tsang et al. (2025)	China	Retrospective cohort study	25,586	5764	CHARLS	Community	66.76 ± 5.59	Female 49.34% Male 50.66%	8
Wang et al. (2023)	China	Retrospective cohort study	2312	1466	CHARLS	Community	69.14 ± 1.84	Female 57.37% Male 42.63%	8
Xie et al. (2023)	China	Retrospective cohort study	4910	3646	CHARLS	Community	66.0^a^	Female 50.30% Male 49.70%	8
Yazawa et al. (2025)	Japan	Prospective cohort study	8576	2033	Iwanuma Study	Community	71.9 ± 5.3	Female 43.4% Male 56.6%	7
Yu et al. (2025)	Australia/USA	Prospective cohort study	19,114	12,029	ASPREE	Community	75.1 ± 4.2	Female 55% Male 45%	7
Zhou et al. (2025)	China	Retrospective cohort study	16,954	1666	CLHLS	Community	73.97 ± 11.4	Female 51.4% Male 48.6%	7

*Note*: not applicable.

Abbreviations: ACT, Adult Changes in Thought; AIBL, Australian Imaging, Biomarkers and Lifestyle; ASPREE, ASPirin in Reducing Events in the Elderly; CHARLS, China Health and Retirement Longitudinal Study; CLHLS, Chinese Longitudinal Healthy Longevity Survey; Duke EPESE, Duke Established Populations for Epidemiologic Studies of the Elderly; ELSA, English Longitudinal Study of Ageing; HRS, Health and Retirement Study; KLoSA, Korean Longitudinal Study of Aging; MHAS, Mexican Health and Aging Study; SHARE, Survey of Health, Ageing and Retirement in Europe; UK, United Kingdom; USA, United States.

^a^ Did not report SD.

### 3.1 Basic Characteristics of the Study Inclusion

Samples investigated ranged in size from 423 to 47,010 participants (median = 3428) and mainly comprised females (*M* = 53.5%, Standard Deviation [SD] = 5.3%), with a median age of 69.4 years. Additionally, Liu et al. (Liu et al. 2025) did not report mean age, and their study was excluded from age calculations. Ran et al. (2024) reported separate statistics for three countries (United States, United Kingdom, and Mexico); the China subsample was excluded due to mean baseline age < 60 years. All of the 21 included studies reported that data were best fit by models with more than one trajectory group. Between 2 and 13 trajectory subgroups were identified across studies, yielding 26 independent observations after accounting for within‐study stratification by sex, poverty status, and country. The majority of studies (*n* = 8) identified four trajectory categories, while six studies identified three categories, two studies identified two categories, and five studies identified five or more categories (Yazawa et al. [2025] with 5, Montagnier et al. [2014] with 6, H. Lee, Park, et al. [2017] with 8, and Ran et al. [2024] with 13 across three countries). No single study identified more than five trajectory subgroups in any one country. Data quality was assessed by the NOS (Stang 2010); publications ranged in score from 6 to 8 (mean [SD] = 7.6 [0.6]). Table 1 provides a summary of all included publications.

### 3.2 Study Design and Trajectory Identification Methods

Table 2 shows that 21 studies spanned a wide range of follow‐up durations, with a median follow‐up of 9 years (range 4–20 years) and a median number of follow‐up visits of 4 (range 3–9). Most studies (*n* = 12) employed a prospective cohort design, while the remainder were retrospective cohorts (*n* = 9). Follow‐up intervals ranged from 6 months to 5 years, reflecting variations in the frequency with which studies captured the dynamic changes in depressive symptoms.

**TABLE 2:** Details of depressive symptom trajectory studies included in the review.

Author/publish year	Follow‐up times/duration(year)	Depression symptom assessment tool	Analytical method	Number of tracks	Specific trajectory (*n*, %)
Bao et al. (2023)	4 times/7 years (2011–2018)	CES‐D 10 items	GBTM	4	Persistent low depressive symptoms (*n* = 830, 38.13%) Increasing (*n* = 450, 20.67%) Decreased (*n* = 514, 23.61%) Persistent high (*n* = 383, 17.59%)
Borza et al. (2022)	4 times/5 years (2012–2017)	CSDD	GMM	4	Persistent mild symptoms group (*n* = 214, 32.6%) Persistent moderate (*n* = 333, 50.8%) Increasing (*n* = 33, 5.1%) Severe but decreasing symptoms group (*n* = 76, 11.6%)
Cermakova and Csajbók (2023)	8 times/16 years (2004–2020)	EURO‐D 12 items	GMM	4	Constantly low depressive symptoms (*n* = 33,969, 72.3%) Decreasing (*n* = 5595, 11.9%) Increasing (*n* = 5574, 11.9%) Constantly high (*n* = 1872, 4.0%)
de la Torre‐Luque et al. (2019)	6 times/10 years (2002–2012)	CES‐D 8 items	LCMM	3	Low symptom class (*n* = 9846, 74.57%) Moderate (*n* = 2375, 17.99%) High (*n* = 982, 7.44%)
(Galatzer‐Levy and Bonanno 2014)	5 times/10 years (1994–2004)	CES‐D 9 items	LGMM	4	Resilient (*n* = 1466, 68.3%) Chronic depression (*n* = 301, 14.0%) Emerging depression (*n* = 234, 10.9%) Depressed‐improved (*n* = 146, 6.8%)
Holmes et al. (2018)	5 times/6 years (2006–2012)	HADS‐Depression	LGMM	2	Low/stable (*n* = 346, 81.8%) Chronically elevated (*n* = 77, 18.2%)
Iob et al. (2023)	8 times/14 years (2002–2016)	CES‐D 8 items	GBTM	3	Persistent low depressive symptoms (*n* = 1560, 45.5%) Moderate increasing (*n* = 1549, 45.2%) Persistent high (*n* = 319, 9.3%)
Kuchibhatla et al. (2012)	4 times/10 years (1986–1996)	CES‐D 20 items	GGMM	4	Persistent low (*n* = 3043, 76.6%) Increasing (*n* = 397, 10.0%) Decreasing (*n* = 318, 8.0%) Persistent high (*n* = 215, 5.4%)
Kumar et al. (2021)	6 times/10 years (2005–2015)	CES‐D 10 items	GBTM	4	Male Minimal (*n* = 741, 51.3%) Decreasing (*n* = 287, 19.9%) Increasing (*n* = 253, 17.5%) Persistent (*n* = 164, 11.3%) Female Minimal (*n* = 981, 46.8%) Decreasing (*n* = 139, 6.6%) Increasing (*n* = 683, 32.5%) Persistent (*n* = 293, 14.0%)
H. Lee, Park, et al. (2017)	4 times/6 years (2006–2012 years)	CES‐D 10 items	LCGA	8	Poor High to moderate (*n* = 212, 8.6%) Slightly increasing (*n* = 1086, 44.1%) Stable low (*n* = 905, 36.8%) Stable high (*n* = 160, 6.5%) Steeply increasing (*n* = 98, 4.0%) Non poor High to moderate (*n* = 88, 5.3%) Steeply increasing (*n* = 192, 11.5%) Stable low (*n* = 1388, 83.2%
Liang et al. (2020)	3 times/4 years (2011–2015)	CES‐D 10 items	GMM	4	Stably low depression trajectory (*n* = 1171, 59.03%) Stably high (*n* = 163, 8.2%) Increasing (*n* = 302, 15.24%) Decreasing (*n* = 348, 17.52%)
Liu et al. (2025)	5 times/10 years (2011–2020)	CES‐D 10 items	LGMM	3	Stable low level (*n* = 1405, 76.7%) Anterior high level (*n* = 227, 12.4%) Posterior high level (*n* = 200, 10.9%)
Montagnier et al. (2014)	9 times/20 years (1987–2007)	CES‐D 20 items	GBTM	6	Male Never depressed (*n* = 697, 65.0%) Emerging depression (*n* = 292, 28.0%) Increasing depression (*n* = 70, 7.0%) Female Never depressed (*n* = 863, 56.0%) Rising subclinical (*n* = 509, 33.0%) Persistent depression (*n* = 159, 11.0%)
Pan and Cao (2023)	4 times /7 years (2011–2018)	CES‐D 10 items	dual—trajectory model	3	No depression (*n* = 3383, 59.0%) low depression (*n* = 1812, 31.6%) high depression (*n* = 539, 9.4%)
Ran et al. (2024)	USA: 4 times/8 years (2012–2020) UK: 4 times/9 years (2012–2021) Mexico: 3 times/9 years (2012–2021)	USA, UK: CES‐D 8 items Mexico: CES‐D 9 items	GBTM	USA, UK: 4 Mexico: 5	USA: Low (*n* = 6398, 67.2%) Mild (*n* = 1517, 15.9%) Increasing‐decreasing (*n* = 698, 7.3%) Decreasing‐increasing (*n* = 906, 9.5%) UK: Low (*n* = 3475, 70.4%) Mild (*n* = 772, 15.6%) Increasing‐decreasing (*n* = 280, 5.7%) Decreasing‐increasing (*n* = 409, 8.3%) Mexico: Low (*n* = 2175, 43.3%) Mild (*n* = 916, 18.2%) Increasing‐decreasing (*n* = 742, 14.8%) Decreasing‐increasing (*n* = 879, 17.5%) Decreasing (*n* = 312, 6.2%)
Tsang et al. (2025)	5 times/9 years (2011–2020)	CES‐D 10 items	LCGA	3	Low‐stable (*n* = 3338, 57.91%) Moderate‐increasing (*n* = 1854, 32.17%) High‐increasing (*n* = 572, 9.92%)
Wang et al. (2023)	3 times/4 years (2011–2015)	CES‐D 10 items	GMM	2	Persistently low symptoms (*n* = 847, 57.78%) High but decreasing symptoms (*n* = 619, 42.22%)
Xie et al. (2023)	4 times/7 years (2011–2018)	CES‐D 10 items	GMM	4	Low and stable (*n* = 2322, 63.69%) Increasing (*n* = 609, 16.7%) Decreasing (*n* = 449, 12.31%) High and stable (*n* = 266, 7.3%)
Yazawa et al. (2025)	4 times/9 years (2010–2020)	GDS 15 items	GBTM	5	Stably not depressed (*n* = 252, 12.4%) Increased after the disaster (*n* = 289, 14.2%) Decreased after the disaster (*n* = 179, 8.8%) Persistent mild depressive symptoms (*n* = 571, 28.1%) Persistent severe depressive symptoms (*n* = 742, 36.5%)
Yu et al. (2025)	6 times/7 years (2010–2017)	CES‐D 10 items	LCMM	4	Nondepressed (*n* = 5533, 46.0%) Subthreshold depression (*n* = 4631, 38.5%) Persistent depression (*n* = 1083, 9.0%) Emerging depression (*n* = 782, 6.5%)
Zhou et al. (2025)	4 times/10 years (2008–2018)	CES‐D 10 items	GBTM	3	Low (*n* = 275, 16.5%) Moderate (*n* = 1171, 70.3%) High progressive (*n* = 220, 13.2%)

*Note*: Percentages for Ran et al. subgroups are calculated relative to each country's analytic sample (USA: *n* = 9519; UK: *n* = 4936; Mexico: *n* = 5024). The China subsample (*n* = 2520) was excluded due to mean baseline age < 60 years and is not listed in this table.

Abbreviations: CES‐D, Center for Epidemiological Survey Depression scale; CSDD, Cornell Scale for Depression in Dementia; EURO‐D, European Union initiative to compare symptoms of depression in 14 European centers; GBTM, Group‐Based Trajectory Model; GDS, Geriatric depression scale; GGMM, Generalized Gaussian mixture model; GMM, Growth Mixture Model; LCGA, Latent Class Growth Analysis; LCMM, Latent Class Mixed Model; LGMM, Latent Growth Mixture model.

Ten different depressive symptom assessment tools were used across the studies, with the CES‐D (Center for Epidemiologic Studies Depression Scale) being the most frequently employed (*n* = 17). However, significant version differences existed, including 8‐item, 9‐item, 10‐item, and 20‐item versions. Other instruments included the Cornell Scale for Depression in Dementia (CSDD, *n* = 1), EURO‐D (*n* = 1), Geriatric Depression Scale (GDS, *n* = 1), and Hospital Anxiety and Depression Scale (HADS, *n* = 1). This measurement heterogeneity suggests that different studies may capture distinct dimensions of depressive symptoms (e.g., CES‐D emphasizes affective symptoms and CSDD emphasizes cognitive‐behavioral symptoms), necessitating consideration of tool specificity when interpreting trajectory categories.

*Analytical methods and number of trajectory categories*: The latent variable mixture models employed across studies included GBTM (Group‐Based Trajectory Model, *n* = 8), GMM (*n* = 8), LCMM (Latent Class Mixed Model, *n* = 4), and LCGA (*n* = 2). The median number of trajectory categories identified per study was 4 (range: 2–13 categories). Notably, H. Lee, Park, et al. (2017) identified eight trajectory subgroups (stratified by poverty status: five classes in the poor group and three in the nonpoor group), Montagnier et al. (2014) identified six classes (stratified by gender: three classes each for males and females), and Holmes et al. (2018) and J. Y. Wang et al. (2023) identified only two trajectory categories, reflecting the impact of model selection, stratification strategies, and population heterogeneity on trajectory subclassification.

Original studies lacked standardized naming conventions for trajectories. For example, the “Low symptom stable” trajectory was labeled as “Persistent low” (Bao et al. 2023) “Resilient” (Galatzer‐Levy and Bonanno 2014), “Never depressed” (Montagnier et al. 2014), or “Low/stable” (Holmes et al. 2018). Similarly, the “high symptom” trajectory was labeled as “Persistent high,” “Chronic Depression,” “High but decreasing,” or “High and increasing,” among others. This naming confusion highlights the necessity for standardized reclassification when comparing across studies (see Table 3).

**TABLE 3:** Modifications of depressive symptom trajectory labels and cutoff scores.

Study	Name of original trajectories	Beginning score	Final score	Trend	Assessment tools	Symptom level	Type
Bao et al. (2023)	Persistent low	3.0	4.0	Stable	CES‐D 10: 10 items scored 0 ‐3 (range 0–30)	Low	Low‐Stable
	Increasing	6.0	12.0	Increasing		High	High‐Increasing
	Decreasing	11.0	7.0	Decreasing		Low	Low‐Decreasing
	Persistent high	15.0	17.0	Stable		High	High‐Stable
Borza et al. (2022)	Persistent Mild	2.2	3.5	Stable	CSDD: 19 items scored 0–2 (range 0–38)	Low	Low‐Stable
	Persistent Moderate	6.7	7.5	Stable		Low	Low‐Stable
	Increasing	5.8	16.0	Increasing		High	High‐Increasing
	Severe but Decreasing	15.2	11.0	Decreasing		Moderate	Moderate‐Decreasing
Cermakova and Csajbók (2023)	Persistently low	1.5	1.7	Stable	EURO‐D: 12 items scored 0–1 (range 0–12)	Low	Low‐Stable
	Decreasing	5.2	3.8	Decreasing		Moderate	Moderate‐Decreasing
	Increasing	2.3	4.8	Increasing		High	High‐Increasing
	Persistently High	6.5	7.0	Stable		High	High‐Stable
de la Torre‐Luque et al. (2019)	Low	1.5	2.5	Increasing	CES‐D 8: 8 items scored 0–1 (range 0–8)	Moderate	Moderate‐Increasing
	Moderate	3.0	4.0	Increasing		High	High‐Increasing
	High	6.0	5.0	Decreasing		High	High‐Decreasing
Galatzer‐Levy and Bonanno (2014)	Resilient	1.6	1.7	Stable	CES‐D 9: 9 items scored 0—1 (range 0–9)	Low	Low‐Stable
	Chronic Depression	5.5	6.3	Stable		High	High‐Stable
	Emerging Depression	2.4	4.7	Inverted U‐shaped		High	High‐Inverted U‐shaped
	Depressed‐Improved	1.9	3.7	Inverted U‐shaped		High	High‐Inverted U‐shaped
Holmes et al. (2018)	Low/stable	1.9	1.9	Stable	HADS: 7 items scored 0–2 (range 0–14)	Low	Low‐Stable
	Chronically elevated	5.3	5.3	Stable		Moderate	Moderate‐Stable
Iob et al. (2023)	Persistent low	0.4	0.4	U‐shaped	CES‐D 8: 8 items scored 0—1 (range 0–8)	Low	Low‐U‐shaped
	Moderate increasing	1.6	1.7	Stable		Low	Low‐Stable
	Persistent high	4.3	4.2	Inverted U‐shaped		High	High‐Inverted U‐shaped
Kuchibhatla et al. (2012)	Persistent low	1.5	2.0	Stable	modified CES‐D:20 items scored 0–2 (range 0–16)	Low	Low‐Stable
	Increasing	3.0	9.0	Increasing		High	High‐Increasing
	Decreasing	8.3	6.0	Decreasing		Moderate	Moderate‐Decreasing
	Persistent high	12.0	10.0	Stable		High	High‐Stable
Kumar et al. (2021)	Male						
	Minimal	1.0	1.5	Stable	CES‐D 10: 10 items scored 0‐3(range 0‐30)	Low	Low‐Stable
	Decreasing	5.0	2.8	Decreasing		Low	Low‐Decreasing
	Increasing	2.0	7.0	Increasing		Low	Low‐Increasing
	Persistent	9.0	9.5	Stable		Moderate	Moderate‐Stable
	Female						
	Minimal	1.5	2.0	Stable		Low	Low‐Stable
	Decreasing	7.5	2.0	Decreasing		Low	Low‐Decreasing
	Increasing	4.0	5.5	Increasing		Low	Low‐Increasing
	Persistent	9.5	10.0	Stable		Moderate	Moderate‐Stable
H. Lee, Park, et al. (2017)	Poor						
	High to Moderate	19.0	10.0	Decreasing	CES‐D 10: 10 items scored 0–3 (range 0–30)	Modertate	Moderate‐Decreasing
	Slightly increasing	8.0	10.0	Stable		Moderate	Moderate‐Stable
	Stable low	6.0	6.0	Stable		Low	Low‐Stable
	Stable high	18.0	19.0	Stable		High	High‐Stable
	Steeply increasing	8.0	22.0	Increasing		High	High‐Increasing
	Nonpoor						
	High to Moderate	18.0	8.0	Decreasing		Moderate	Moderate‐Decreasing
	Steeply increasing	9.0	18.0	Increasing		High	High‐Increasing
	Stable low	6.0	7.0	Stable		Low	Low‐Stable
Liang (2020)	Stably low	6.0	6.0	Stable	CES‐D 10: 10 items scored 0–3 (range 0–30)	Low	Low‐Stable
	Stably high	20.2	20.0	Stable		High	High‐Stable
	Increasing	9.5	16.0	Increasing		Moderate	Moderate‐Increasing
	Decreasing	15.9	8.0	Decreasing		Moderate	Moderate‐Decreasing
Liu et al. (2025)	Stable low	6.5	7.0	Stable	CES‐D 10: 10 items scored 0–3 (range 0–30)	Low	Low‐Stable
	Anterior high	17.5	15.0	Decreasing		High	High‐Decreasing
	Posterior high	8.8	18.0	Increasing		High	High‐Increasing
Montagnier et al. (2014)	Male						
	Never depressed	4.0	12.0	Increasing	CES‐D 20: 20 items scored 0–3 (range 0–60)	Low	Low‐Increasing
	Emerging depression	10.0	28.5	Increasing		High	High‐Increasing
	Increasing depression	21.0	30.0	Increasing		High	High‐Increasing
	Female						
	Never depressed	7.0	12.0	Increasing		Low	Low‐Increasing
	Rising subclinical	15.0	20.0	Increasing		Moderate	Moderate‐Increasing
	Persistent depression	27.0	27.0	Stable		High	High‐Stable
Pan and Cao (2023)	No depression	5.7	7.5	Increasing	CES‐D 10: 10 items scored 0–3 (range 0–30)	Low	Low‐Increasing
	Low depression	12.4	12.6	Increasing		High	High‐Increasing
	High depression	18.6	18.2	Decreasing		High	High‐Decreasing
Ran et al. (2024)	USA				CES‐D 8: 8 items scored 0–1 (range 0–8)		
	Low	0.3	0.5	Stable		Low	Low‐Stable
	Mild	1.4	1.8	Stable		Low	Low‐Stable
	Increasing‐decreasing	1.9	2.0	Inverted U‐shaped		Low	Low‐Inverted U‐shaped
	Decreasing‐increasing	3.1	4.1	U‐shaped		High	High‐U‐shaped
	UK				CES‐D 8: 8 items scored 0–1 (range 0–8)		
	Low	0.4	0.5	Stable		Low	Low‐Stable
	Mild	1.2	2.0	Increasing		Low	Low‐Increasing
	Increasing‐decreasing	1.7	1.8	Inverted U‐shaped		Low	Low‐Inverted U‐shaped
	Decreasing‐increasing	3.0	4.5	U‐shaped		High	High‐U‐shaped
	Mexico				CES‐D 9: 9 items scored 0–1 (range 0–9)		
	Low	1.2	1.2	Stable		Low	Low‐Stable
	Mild	2.2	2.8	Increasing		Moderate	Moderate‐Increasing
	Increasing‐decreasing	4.0	3.3	Inverted U‐shaped		Moderate	Moderate‐Inverted U‐shaped
	Decreasing‐increasing	4.2	5.6	U‐shaped		High	High‐U‐shaped
	Decreasing	6.0	2.0	Decreasing		Low	Low‐Decreasing
Tsang et al. (2025)	Low‐Stable	5.4	6.5	Stable	CES‐D 10: 10 items scored 0–3 (range 0–30)	Low	Low‐Stable
	Moderate‐Increasing	11.4	13.5	Increasing		High	High‐Increasing
	High‐Increasing	18.3	19.5	Increasing		High	High‐Increasing
Wang et al. (2023)	Persistently low	8.2	8.0	Stable	CES‐D 10: 10 items scored 0–3 (range 0–30)	Moderate	Moderate‐Stable
	High but decreasing	18.0	14.1	Decreasing		High	High‐Decreasing
Xie et al. (2023)	Low and stable	5.8	5.7	Stable	CES‐D 10: 10 items scored 0–3 (range 0–30)	Low	Low‐Stable
	Increasing	9.4	11.9	Increasing		Moderate	Moderate‐Increasing
	Decreasing	18.0	10.5	Decreasing		Moderate	Moderate‐Decreasing
	High and stable	19.6	21.3	Stable		High	High‐Stable
Yazawa et al. (2025)	Stably not depressed	0.9	1.0	Stable	GDS‐15: 15 items scored 0‐1 (range 0–15)	Low	Low‐Stable
	Increased after the disaster	0.9	3.0	Increasing		Low	Low‐Increasing
	Decreased after the disaster	3.0	1.0	Decreasing		Low	Low‐Decreasing
	Persistent mild	3.0	3.2	Stable		Low	Low‐Stable
	Persistent severe	5.9	6.0	Stable		High	High‐Stable
Yu et al. (2025)	Nondepressed	1.5	2.5	Stable	CES‐D 10: 10 items scored 0–3 (range 0–30)	Low	Low‐Stable
	Subthreshold depression	4.5	5.5	Stable		Low	Low‐Stable
	Emerging depression	1.0	6.5	Increasing		Low	Low‐Increasing
	Persistent depression	8.0	9.5	Stable		Moderate	Moderate‐Stable
Zhou et al. (2025)	Low	4	3	Stable	5‐item scale of depressive symptom: 5 items scored 0–4 (range 0–20)	Low	Low‐Stable
	Moderate	6	6	Stable		Moderate	Moderate‐Stable
	High Progressive	8	9	Increasing		High	High‐Increasing

*Note*: Low/Moderate/High, symptom severity; Increasing/Decreasing/Stable, trajectory trend; Inverted U‐shaped, increasing‐decreasing; U‐shaped, decreasing‐increasing.

Among studies reporting trajectory patterns (*n* = 21), stable trajectories (Stable) were most prevalent (*n* = 20), followed by increasing trajectories (Increasing, *n* = 18) and decreasing trajectories (Decreasing, *n* = 16). Nonlinear trajectories (U‐shaped or Inverted U‐shaped) were identified in three studies (five observations when stratified subsamples were counted separately), though naming and definitions varied considerably. For example, Ran et al. (2024) identified “increasing‐decreasing” and “decreasing‐increasing” patterns in a multinational sample across three countries (United States, United Kingdom, and Mexico), while Galatzer‐Levy and Bonanno (2014) identified “emerging‐depression” (Inverted U‐shaped) and “Depressed‐improved” (Inverted U‐shaped).

### 3.3 Patterns of Depressive Symptom Trajectories

Table 3 presents the standardized trajectory categories based on final symptom severity (low, moderate, and high) and dynamic trend (stable, increasing, decreasing, U‐shaped, and inverted U‐shaped). These labels serve as heuristic anchors for cross‐study comparison rather than clinically validated diagnostic categories, given the post hoc nature of the rescaling and the heterogeneity of original instruments. Between‐study heterogeneity was extreme across both dimensions, precluding meaningful statistical pooling. Specific prevalence estimates for each trajectory are detailed in Table 2.

Low‐symptom trajectories represented the modal pattern across included studies, reflecting the predominant distribution of minimal depressive symptoms throughout observation periods. These configurations comprised five distinct subtypes. Stable‐low patterns encompassed Persistently low (Bao et al. 2023), Resilient (Galatzer‐Levy and Bonanno 2014), Never depressed (Montagnier et al. 2014), Stably not depressed (Yazawa et al. 2025), Low and stable (Xie et al. 2023), Persistent low (Iob et al. 2023; Kuchibhatla et al. 2012), Stable low (H. Lee, Park, et al. 2017; Liang 2020; Tsang et al. 2025), No depression (Pan and Cao 2023), Nondepressed (Yu et al. 2025), Subthreshold depression (Yu et al. 2025), and Low (Zhou et al. 2025) trajectories, representing individuals who maintained minimal symptom burdens continuously. Notably, several trajectories originally labeled as “moderate” or “subthreshold” in their source studies, such as Persistent Moderate in nursing home residents (Borza et al. 2022) and Subthreshold depression in community dwellers (Yu et al. 2025), fell below the rescaled CES‐D threshold of 16 upon standardization, underscoring the metric dependency of original labels. Decreasing‐low subtypes captured participants demonstrating substantial remission from initially elevated states to below the screening threshold, exemplified by Post‐disaster decrease (Yazawa et al. 2025), Decreasing classifications in male and female community cohorts (Kumar et al. 2021), and the Mexico subsample of a multinational study (Ran et al. 2024). Low‐increasing trajectories, characterized by subclinical symptom escalation that remained below the moderate threshold, were identified in community samples (Kumar et al. 2021; Montagnier et al. 2014; Pan and Cao 2023; Yu et al. 2025) and in the UK subsample of a multinational cohort (Ran et al. 2024). Additionally, nonlinear configurations with peak severity below 16 CES‐D equivalents were observed, including U‐shaped (Iob et al. 2023) and inverted U‐shaped (Ran et al. 2024) patterns. Prevalence estimates for the combined low‐symptom stratum ranged from 16.5% in rural Chinese cohorts (Zhou et al. 2025) to 84.5% in high‐functioning community cohorts (Holmes et al. 2018; Yu et al. 2025), with marked variability reflecting population characteristics and instrument‐specific score distributions. In sum, low‐symptom trajectories were the modal pattern across all settings, yet their prevalence varied considerably depending on baseline vulnerability and environmental stressors.

Moderate‐symptom trajectories exhibited the greatest cross‐study heterogeneity, constituting anywhere from negligible proportions to moderate majorities depending on demographic context. This stratum comprised three temporal patterns. Moderate‐Stable configurations maintaining persistent intermediate symptom levels were identified in studies employing the HADS‐D (Holmes et al. 2018), CES‐D 10‐item (H. Lee, Park, et al. 2017; J. Y. Wang et al. 2023; Yu et al. 2025; Zhou et al. 2025), and CES‐D 8‐item (Kumar et al. 2021) instruments, with prevalence estimates ranging from 9.0% (Yu et al. 2025) to 70.3% (Zhou et al. 2025). Moderate‐Increasing trajectories demonstrated progressive worsening from subclinical baselines, exemplified by de la Torre‐Luque et al. (2019), Montagnier et al. (2014), and nonlinear moderate‐symptom configurations in multinational cohorts (Ran et al. 2024). Moderate‐Decreasing patterns indicated substantial improvement from initially elevated presentations to sustained intermediate levels, as observed in Borza et al. (2022), Cermakova and Csajbók (2023), Kuchibhatla et al. (2012), H. Lee, Park, et al. (2017), Liang (2020), and Xie et al. (2023), with rescaled final scores falling within the 16–24 moderate range. Collectively, moderate‐symptom trajectories exhibited the greatest cross‐study inconsistency, suggesting that intermediate symptom states may be unstable or context‐dependent rather than universally identifiable.

High‐symptom trajectories comprised high‐risk subgroups warranting clinical attention, with shape‐based subtyping revealing distinct prognostic profiles. It should be emphasized that these prevalence estimates reflect standardized approximations; the true clinical severity in any given study depends on the original instrument's properties and population norms. Stable‐high patterns indicated chronic persistent depression, ranging from 4.0% in European community samples (Cermakova and Csajbók 2023) to 36.5% in post‐disaster settings (Yazawa et al. 2025). Increasing‐high trajectories encompassing emergent and escalating configurations (Bao et al. 2023; Borza et al. 2022; Cermakova and Csajbók 2023; de la Torre‐Luque et al. 2019; Kuchibhatla et al. 2012; H. Lee, Park, et al. 2017; Liang 2020; Liu et al. 2025; Pan and Cao 2023; Tsang et al. 2025; Zhou et al. 2025), and nonlinear high‐symptom configurations (Galatzer‐Levy and Bonanno 2014; Montagnier et al. 2014), with prevalence estimates varying widely across demographic contexts. Decreasing‐high patterns demonstrated spontaneous remission from severe baseline presentations, exemplified by de la Torre‐Luque et al. (2019), Liu et al. (2025), Pan and Cao (2023), and J. Y. Wang et al. (2023), all characterized by rescaled final scores remaining at or above 24 despite downward trends. Nonlinear configurations included Inverted U‐shaped patterns characterized by initial symptom emergence followed by subsequent improvement (Galatzer‐Levy and Bonanno 2014; Iob et al. 2023; Ran et al. 2024) and U‐shaped patterns showing initial decline followed by recurrence (Ran et al. 2024), with classification into symptom strata determined by final severity levels in accordance with the standardized protocol: Inverted U‐shaped trajectories with final scores ≥ 24 fell within the high‐symptom stratum (Galatzer‐Levy and Bonanno 2014; Iob et al. 2023), whereas those with lower final scores were classified as moderate (Ran et al. 2024) or low (Ran et al. 2024) depending on the subsample. Taken together, high‐symptom trajectories were identified in the majority of studies but showed marked variation in prevalence, indicating that chronic or worsening depression in late life is neither universal nor uniformly distributed across populations.

Furthermore, institutional settings (Borza et al. 2022) exhibited a lower proportion of high‐symptom trajectories (5.1% increasing‐high) compared to community post‐disaster settings (Yazawa et al. 2025) (36.5% persistent severe), whereas high‐functioning European samples (Cermakova and Csajbók 2023) nevertheless exhibited clinically significant high‐symptom trajectories alongside predominant low‐symptom patterns. These discrepancies highlight the critical influence of environmental context, functional status, and care setting on depression trajectory distribution.

### 3.4 Sensitivity Analyses

As detailed in Table 4, excluding the largest study (Cermakova and Csajbók (2023); *n* = 47,010) resulted in minimal change to the overall pattern, with the median prevalence remaining within similar ranges (14.3% vs. 13.2%) and the full distribution spanning 0%–42.2% across the remaining 25 observations.

**TABLE 4:** Sensitivity analyses for the prevalence of high‐symptom trajectories among older adults.

Analysis	*k*	Median (%)	IQR (%)	Range (%)	Key studies included
Overall	26	14.3	8.6–33.6	0.0–42.2	All eligible observations
Excluding largest study	25	13.2	8.3–31.7	0.0–42.2	(Cermakova and Csajbók 2023)
Publication period					
2010–2020	9	15.4	10.5–25.4	0.0–34.2	(Kuchibhatla et al. 2012; Galatzer‐Levy and Bonanno 2014; Montagnier et al. 2014; H. Lee, Park, et al. 2017; Holmes et al. 2018; de la Torre‐Luque et al. 2019; Liang 2020)
2021–2025	17	13.2	7.3–36.5	0.0–42.2	(Wang et al. 2023; Bao et al. 2023; Yazawa et al. 2025; Ran et al. 2024; Liu et al. 2025; Cermakova and Csajbók 2023; Zhou et al. 2025; Tsang et al. 2025; Ran et al. 2024; Pan and Cao 2023; Iob et al. 2023; Xie et al. 2023; Borza et al. 2022; Yu et al. 2025; Kumar et al. 2021)
Residential setting					
Community	25	13.2	8.3–31.7	0.0–42.2	All studies except Borza (Borza et al. 2022)
Institutional	1	5.0	—	5.0	Borza et al. (Borza et al. 2022)
One per cohort	14	14.3 6.1–28.2	14.3 6.1–28.2	0.0–41.0	(Pan and Cao 2023; Iob et al. 2023; Galatzer‐Levy and Bonanno 2014; H. Lee, Park, et al. 2017; Montagnier et al. 2014; Kumar et al. 2021; Cermakova and Csajbók 2023; Borza et al. 2022; Holmes et al. 2018; Kuchibhatla et al. 2012; Ran et al. 2024; Yazawa et al. 2025; Yu et al. 2025; Zhou et al. 2025)

*Note: k*, number of observations (includes sex‐specific, socioeconomic, and country‐specific subgroups from the same study); IQR, interquartile range (Q1–Q3); High‐symptom trajectories include groups labeled as high‐stable, high‐increasing, persistent high, or severe depression trajectories in original studies. Institutional studies involved care facilities for older adults with dementia; For Ran et al., country‐specific prevalence estimates in Table 4 were recalculated based on each national subsample denominator to ensure comparability with other single‐country studies.

Stratification by publication period revealed some variation across cohorts. Among the nine observations (seven studies) published between 2010 and 2020, the median prevalence of high‐symptom trajectories was 15.4% (interquartile range 10.5%–25.4%), while the 17 observations (14 studies) from 2021 to 2025 showed a median of 13.2% (interquartile range 7.3%–36.5%). Despite differences in central tendency, the ranges overlapped substantially between the two periods.

To address potential nonindependence from overlapping cohorts, we conducted a one‐per‐cohort sensitivity analysis. For each overlapping database, we retained the study with the longest follow‐up or largest analytical sample (e.g., Pan and Cao [2023] for CHARLS; Iob et al. [2023] for ELSA; and Ran et al. [2024) for HRS). This restricted analysis (*k* = 14) yielded a median high‐symptom prevalence of 14.3% (IQR 6.1%–28.2%; range 0.0%–41.0%), closely aligned with the full‐sample estimate (14.3%). The distribution narrowed moderately at the lower end but retained comparable central tendency and upper‐bound variability, suggesting that the observed cross‐study heterogeneity is not an artifact of cohort duplication.

Comparisons by residential environment indicated that community‐based studies (*k* = 25) reported a median prevalence of 13.2% (range 0%–42.2%), whereas the single institutional study (Borza et al. (2022)) showed a prevalence of 5.0%. While the institutional estimate fell within the lower spectrum, the overall distributions overlapped partially, as community‐based investigations themselves varied widely from 0% to 42.2%.

These analyses suggest that the marked variability in high‐symptom trajectory prevalence represents genuine dispersion across populations rather than artifact from single large studies, publication periods, residential settings, or overlapping cohorts. The consistent identification of distinct trajectory groups across diverse contexts supports the validity of the latent class approach.

### 3.5 Predictors of Older Depressive Symptom Trajectories

As shown in Table 5, among the 21 studies examining baseline predictors of high‐symptom trajectory membership, three consistent risk factors emerged with replication in three or more independent samples. Female sex represented the most prevalent discriminator, with significant associations in seven studies, odds ratios (ORs) or relative risk ratios (RRRs) ranging from 1.43 to 3.98 (Bao et al. 2023; de la Torre‐Luque et al. 2019; Holmes et al. 2018; Kuchibhatla et al. 2012; Pan and Cao 2023; Tsang et al. 2025; Xie et al. 2023). Physical function impairment, encompassing activities of daily living (ADL) or instrumental activities of daily living (IADL) limitations, emerged as the strongest quantitative predictor, with significant effects in five studies, ORs or RRRs ranging from 1.09 to 2.04 (Bao et al. 2023; de la Torre‐Luque et al. 2019; Kuchibhatla et al. 2012; Pan and Cao 2023; J. Y. Wang et al. 2023). Comorbidity or multimorbidity demonstrated consistent risk associations across three studies, ORs 1.44–1.84 (Borza et al. 2022; de la Torre‐Luque et al. 2019; Pan and Cao 2023).

**TABLE 5:** Significant baseline predictors of the highest symptom trajectory in geriatric depression: Results from multivariate analysis.

Category	Predictor	Number of studies	Sample size range	Effect direction and range	Nature of evidence	References
Physical function and health	Physical function impairment/ADL or IADL limitations	5	*n* = 1466–13,203	OR/RRR: 1.09–2.04; aOR: 1.32–1.35; all > 1	Risk factor	(Bao et al. 2023; de la Torre‐Luque et al. 2019; Pan and Cao 2023; Wang et al. 2023; Kuchibhatla et al. 2012)
	Comorbidity/multimorbidity	3	*n* = 655–13,203	OR: 1.44–1.84; RRR: 1.56; all > 1	Risk factor	(Borza et al. 2022; de la Torre‐Luque et al. 2019; Pan and Cao 2023)
	Pain	2	*n* = 655–1466	OR: 1.25–1.39; all > 1	Risk factor	(Borza et al. 2022; Wang et al. 2023)
	Cognitive impairment	2	*n* = 423–3973	OR: 1.11; RRR: 2.7; all > 1	Risk factor	(Kuchibhatla et al. 2012; Holmes et al. 2018)
	Poor self‐rated health	1	*n* = 3973	OR: 2.69 (2.16, 3.33)	Risk factor	(Kuchibhatla et al. 2012)
	Dyspnoea	2	*n* = 1832–2590	OR: 2.8–6.0; all > 1	Risk factor	Liu et al. 2025; Montagnier et al. 2014)
	Sarcopenia/obesity‐related (D/AO, D/NAO)	1	*n* = 1832	OR: 1.53; RR: 2.01–2.15; all > 1	Risk factor	(Liu et al. 2025)
	TBI with LOC (males)	1	*n* = 3541	OR: 1.51 (1.01, 2.27)	Risk factor	(Kumar et al. 2021)
Sociodemographic and psychosocial factors	Female sex	7	*n* = 423–13,203	OR/RRR: 1.43–3.98; OR: 1.91; all > 1	Risk factor	(Bao et al. 2023; de la Torre‐Luque et al. 2019; Holmes et al. 2018; Kuchibhatla et al. 2012; Pan and Cao 2023; Tsang et al. 2025; Xie et al. 2023)
	Low education level	4	*n* = 3973–47,010	OR: 0.85–0.95 (per year increase); RRR: 0.26; all < 1	Protective factor	(de la Torre‐Luque et al. 2019; Kuchibhatla et al. 2012; Pan and Cao 2023; Cermakova and Csajbók 2023)
	Widowed (females)	1	*n* = 5734	OR: 0.4 (0.3, 0.7); < 1	Protective factor	(Pan and Cao 2023)
	Rural residence	1	*n* = 5734	OR: 1.93 (1.46, 2.55)	Risk factor	(Pan and Cao 2023)
	Low income/low socioeconomic status	1	*n* = 5764	RRR: 1.48–5.04; all > 1	Risk factor	(Tsang et al. 2025)
	Household crowding in childhood	1	*n* = 47,010	OR: 1.09–1.12 (1.06, 1.17)	Risk factor	(Cermakova and Csajbók 2023)
	Childhood adverse experiences (ACEs)	1	*n* = 3428	OR: 1.44 (1.30, 1.60)	Risk factor	(Iob et al. 2023)
	Negative wealth shock	1	Multi‐country, *n* > 10,000	OR: 2.01–2.41; all > 1	Risk factor	(Ran et al. 2024)
	Smaller social network/decreased social participation	2	*n* = 3973–4129	OR: 0.96; RRR: 1.76–4.01; mixed	Risk/Protective factor	(Kuchibhatla et al. 2012; H. Lee, Park, et al. 2017)
Health behaviors and medical factors	Antidepressant use	2	*n* = 655–2590	OR: 2.18; RRR: 2.7; all > 1	Risk factor	(Borza et al. 2022; Montagnier et al. 2014)
	Benzodiazepine use (females)	1	*n* = 2590	OR: 1.7 (1.1, 2.9)	Risk factor	(Montagnier et al. 2014)
	Polypharmacy (≥ 5 non‐psychotropic drugs, females)	1	*n* = 2590	OR: 2.0 (1.2, 3.3)	Risk factor	(Montagnier et al. 2014)
	Alcohol use (males, military employment)	1	*n* = 3541	OR: 1.54 (1.03, 2.31)	Risk factor	(Kumar et al. 2021)
Social and environmental resources	Larger social network/higher social interaction	1	*n* = 3973	OR: 0.96–0.98; all < 1	Protective factor	(Kuchibhatla et al. 2012)
	High housing quality	1	*n* = 1984	RRR: 0.795; < 1	Protective factor	(Liang 2020)
	Public health insurance	1	*n* = 5734	OR: 0.7 (0.53, 0.93)	Protective factor	(Pan and Cao 2023)
	Longevity expectation/economic optimism	1	*n* = 2147	b: −0.017; < 0	Protective factor	(Galatzer‐Levy and Bonanno 2014)
	High plant‐based diet index (PDI)	1	*n* = 1666	OR: 0.344 (0.17, 0.69)	Protective factor	(Zhou et al. 2025)
	Military employment (males, symptom decrease)	1	*n* = 3541	OR: 1.54 (1.03, 2.31)	Protective factor	(Kumar et al. 2021)
Genetic and biological markers	Major depression polygenic score (PGS)	2	*n* = 3428–12,029	OR: 1.4–1.47; all > 1	Risk factor	(Iob et al. 2023; Yu et al. 2025)
	BDNF Val66Met allele carrier (Females)	1	*n* = 423	RRR: 2.7 (1.07, 6.82)	Risk factor	(Holmes et al. 2018)
Trauma and stress‐related	Severe post‐traumatic stress symptoms (PTSS)	1	*n* = 2033	RRR: 4.09–11.8; all > 1	Risk factor	(Yazawa et al. 2025)
	Childhood physical abuse	1	*n* = 5764	RRR: 1.46 (1.12, 1.90)	Risk factor	(Tsang et al. 2025)
	Parental mental illness	1	*n* = 5764	RRR: 2.92 (2.31, 3.69)	Risk factor	(Tsang et al. 2025)
	Poor childhood health	1	*n* = 5764	RRR: 3.43 (2.10, 5.61)	Risk factor	(Tsang et al. 2025)
	History of depressive disorder	2	*n* = 1832–2590	OR: 2.7–4.4; all > 1	Risk factor	(Montagnier et al. 2014; Liu et al. 2025)
	Other psychiatric disorder history (Females)	1	*n* = 2590	OR: 4.1 (1.8, 9.5)	Risk factor	(Montagnier et al. 2014)

*Note*: “All > 1” indicates that all included studies reported effect sizes greater than 1 (increased risk), with ranges showing the lowest and highest estimates; “all < 1” indicates consistent protective effects (decreased risk); “mixed” indicates conflicting directions across studies. Values in parentheses represent 95% confidence intervals where reported in original studies; military employment showed divergent associations depending on baseline symptom status: protective against incident depression but predictive of symptom persistence in already‐depressed individuals (Kumar et al. 2021).

Abbreviations: ACEs, Adverse Childhood Experiences; ADL, Activities of Daily Living; aOR, adjusted Odds Ratio; APOE, Apolipoprotein E; b, unstandardized regression coefficient; BDNF, Brain‐Derived Neurotrophic Factor; CI, confidence interval; D/AO, Dynapenia with Abdominal Obesity; D/NAO, Dynapenia without Abdominal Obesity; IADL, Instrumental Activities of Daily Living; LOC, Loss of Consciousness; OR, Odds Ratio; PDI, Plant‐based Diet Index; PGS, Polygenic Score; PTSS, Post‐Traumatic Stress Symptoms; RRR, Relative Risk Ratio; TBI, Traumatic Brain Injury.

Consistent protective factors were less frequently examined but showed robust inverse associations. Higher educational attainment emerged as the most reliable protective factor, with significant effects in four studies, ORs ranging from 0.26 to 0.95 per year increase (Cermakova and Csajbók 2023; de la Torre‐Luque et al. 2019; Kuchibhatla et al. 2012; Pan and Cao 2023). Larger social network size or higher social interaction frequency demonstrated protective effects in one study, ORs 0.96–0.98 (Kuchibhatla et al. 2012). Factors with inconsistent evidence or limited to single reports spanned multiple domains. Pain (Borza et al. 2022; J. Y. Wang et al. 2023), cognitive impairment (Holmes et al. 2018; Kuchibhatla et al. 2012), dyspnoea (Liu et al. 2025; Montagnier et al. 2014), sarcopenia or obesity‐related measures (Liu et al. 2025), and antidepressant use (Borza et al. 2022; Montagnier et al. 2014) each showed significant risk associations in two studies, except sarcopenia, which was reported in one study. Low income or low socioeconomic status demonstrated risk associations in one study (Tsang et al. 2025), whereas smaller social networks or decreased social participation yielded mixed directional associations across two studies (Kuchibhatla et al. 2012; H. Lee, Park, et al. 2017). Single‐study risk factors encompassed poor self‐rated health (Kuchibhatla et al. 2012), traumatic brain injury with loss of consciousness in males (Kumar et al. 2021), rural residence (Pan and Cao 2023), household crowding in childhood (Cermakova and Csajbók 2023), childhood adverse experiences (Iob et al. 2023), negative wealth shock (Ran et al. 2024), benzodiazepine use (Montagnier et al. 2014), polypharmacy (Montagnier et al. 2014), alcohol use in males (Kumar et al. 2021), severe post‐traumatic stress symptoms (Yazawa et al. 2025), childhood physical abuse (Tsang et al. 2025), parental mental illness (Tsang et al. 2025), poor childhood health (Tsang et al. 2025), history of depressive disorder (Liu et al. 2025; Montagnier et al. 2014), and other psychiatric disorder history (Montagnier et al. 2014). Genetic markers, including major depression polygenic scores (Iob et al. 2023; Yu et al. 2025) and BDNF Val66Met allele carrier status (Holmes et al. 2018), showed significant risk associations in one or two studies.

Protective factors limited to single reports included widowed status in females (Pan and Cao 2023), public health insurance (Pan and Cao 2023), high housing quality (Liang 2020), longevity expectation or economic optimism (Galatzer‐Levy and Bonanno 2014), high plant‐based diet index (Zhou et al. 2025), and military employment in males (Kumar et al. 2021).

It should be noted that several significant associations for female sex (Bao et al. 2023; Pan and Cao 2023; Tsang et al. 2025; Xie et al. 2023), functional limitations (Bao et al. 2023; Pan and Cao 2023; Tsang et al. 2025), and education (Cermakova and Csajbók 2023; de la Torre‐Luque et al. 2019; Kuchibhatla et al. 2012; Pan and Cao 2023) were derived from overlapping cohorts (CHARLS, ELSA, and HRS), so the apparent consistency may partially reflect shared population characteristics rather than independent replication.

Physical health indicators consistently outperformed psychosocial variables in discriminating high‐symptom trajectories, with functional limitations, multimorbidity, and pain forming a coherent cluster of somatic risk factors. Conversely, social and environmental resources, particularly educational attainment and social network integration, demonstrated the most robust protective effects against persistent high symptom burden.

## 4 Discussion

### 4.1 Summary of Key Findings

This systematic review synthesized evidence from 21 longitudinal studies using latent variable mixture models to delineate depressive symptom trajectories in older adults.

Low‐symptom trajectories predominated across studies (range 12.4%–84.5%), typically manifesting as stable patterns reflecting emotional resilience.

High‐symptom trajectories, encompassing chronic‐stable, progressive‐increasing, and remitting‐decreasing subtypes (the latter characterized by final scores remaining at or above 24 despite downward trends)—were identified in 18 of 21 studies, with prevalence ranging markedly from 0% to 42.2%. This range should be interpreted as indicative of plausible dispersion across populations rather than precise global estimates, given the standardized but unvalidated cross‐instrument calibration applied. This marked variation, spanning from 0% in cognitively intact, community‐residing older adults to 42.2% among disabled rural populations, underscores the profoundly context‐dependent nature of late‐life depression burden. Such divergence reflects the dynamic interplay of care settings, physical functional status, and socioeconomic gradients in shaping mental health trajectories, suggesting that uniform prevention strategies may inadequately address the heterogeneous needs of aging populations.

Moderate‐symptom trajectories exhibited substantial cross‐study variability, with some studies identifying over 50% of participants in this category while others observed strictly bimodal distributions.

Risk factors consistently associated with adverse trajectories included female sex, functional limitations and disability, and multimorbidity. Limited evidence also suggested associations for low socioeconomic status (Tsang et al. 2025), pain (Borza et al. 2022; J. Y. Wang et al. 2023), childhood adversity (Iob et al. 2023), and polygenic risk scores (Iob et al. 2023; Yu et al. 2025).

### 4.2 Interpretation in the Context of Existing Evidence

The predominance of the low‐stable trajectory aligns with socioemotional selectivity theory, wherein older adults prioritize emotionally meaningful goals, potentially enhancing emotion regulation and resilience (English and Carstensen 2016; Wen et al. 2022). This pattern demonstrates notable cross‐cultural consistency, replicated across community‐dwelling cohorts in North America, Europe, and Asia (Galatzer‐Levy and Bonanno 2014; Kuchibhatla et al. 2012; Wen et al. 2022; Agustini et al. 2022).

The median prevalence of high‐symptom trajectories (∼14.3%) is broadly consistent with estimates from high‐quality cohort studies employing varied methodologies, such as the “persistent/emergent” classes (15.6%) identified by Agustini et al. (2022) and the “late‐increasing” group (20%) reported by Zhao et al. (2024), reinforcing the validity of this adverse subgroup. While these comparisons are suggestive, they should be interpreted cautiously because the external estimates were derived under each study's original classification scheme rather than the standardized taxonomy used here. Furthermore, the extreme range (0%–42%) highlights that while the existence of high‐symptom trajectories is universal, their burden is highly context‐specific.

Past studies indicated substantial between‐study heterogeneity in prevalence estimates. This variability likely reflects the profound etiological complexity of late‐life depression and unmeasured methodological variations—including differences in population characteristics (e.g., dementia prevalence in institutions vs. community samples), cultural contexts, life‐course exposures, and assessment tool specificities (e.g., CES‐D emphasizing affective symptoms vs. CSDD capturing cognitive‐behavioral manifestations in frail, cognitively impaired populations (Kurlowicz et al. 2002)). Research by Wen et al. (2022) and Mitchell et al. (2021) highlights depression's multifactorial nature spanning neurobiological, clinical, and genetic domains. The convergence of evidence on core trajectory patterns (stable‐low, chronic‐high, and increasing) alongside extreme prevalence variability suggests these universal patterns manifest at different absolute levels depending on unmeasured or uncontrollable study‐level factors. Consequently, our descriptive estimates are best interpreted as indicative of a plausible range within defined populations rather than as precise global point estimates.

### 4.3 Mechanisms and Clinical Implications

It is important to clarify that the following discussion does not propose a novel pathogenic theory; rather, it integrates established biopsychosocial frameworks into the specific context of geriatric depression trajectories to generate a translational, evidence‐based intervention logic. The identified predictors collectively support a dynamic, bidirectional model of late‐life depression, integrating biological, psychological, and social pathways. The body‐to‐mind pathway is evidenced by consistent links between objective physical decline (poor extremity function [Bao et al. 2023; Veronese et al. 2017], sarcopenia [Chen et al. 2020]), chronic pain, inflammation (Beurel et al. 2020; Köhler et al. 2017), and membership in adverse trajectories. Conversely, the mind‐to‐body pathway is evident where depressive symptoms reduce motivation for physical activity (Schuch et al. 2018) and social interaction, potentially exacerbating functional decline and somatic illness, thereby creating a pernicious cycle.

This integrative framework is further enriched by life‐course and psychosocial perspectives drawn from existing literature, where early‐life adversities and gender‐specific social determinants (e.g., unmarried status increasing risk for women [J. Lee, Jang, et al. 2017; Zhai et al. 2024]) exert long‐lasting effects on emotional vulnerability, interacting with genetic liability (Agustini et al. 2022; Stringa et al. 2020) to shape later‐life trajectories. The identification of polygenic risk scores as significant predictors, even in late‐life cohorts, suggests that genetic predisposition continues to influence depression course despite accumulated environmental exposures.

Crucially, the standardized trajectory taxonomy presented in Table 3 enables the linkage of these mechanistic insights to specific subgroups: by distinguishing low‐stable, moderate, and high‐symptom trajectories, clinicians can move beyond a one‐size‐fits‐all approach and match intervention intensity to the actual symptom course rather than to a single cross‐sectional severity score. These findings suggest a tiered, precision intervention strategy: (1) maintenance of current well‐being for the low‐stable majority through community‐based activity programs; (2) targeted preventive monitoring for genetically or socially vulnerable subgroups (e.g., those with childhood adversity or functional decline) (Agustini et al. 2022; Stringa et al. 2020); and (3) intensive multidisciplinary intervention combining physical rehabilitation, pain management, and psychotherapy for those in chronic high‐symptom trajectories (Leontjevas et al. 2013; Meeks et al. 2015).

Notably, past studies indicate that employment may prevent depressive episodes, but it also predicts the persistence of symptoms in those who already have them (Kumar et al. 2021). This emphasizes how crucial it is to offer workplace modifications and mental health support to older employees with depression rather than relying solely on professional interventions.

To enable quantitative meta‐analyses in the future, we propose a minimal common assessment protocol for primary studies examining late‐life depression trajectories: (1) baseline and repeated CES‐D 10‐item or 20‐item assessment at intervals ≤ 2 years; (2) standardized recording of functional status (ADL/IADL), multimorbidity count, and educational attainment as core predictors; and (3) transparent reporting of attrition patterns and sensitivity analyses for missing data. Future studies should also test whether modifiable predictors (physical function and social engagement) identified here differentially predict transition probabilities between trajectory classes, moving beyond cross‐sectional predictor‐trajectory associations.

### 4.4 Limitations

Several limitations must be considered.

(a) The substantial between‐study heterogeneity in measurement tools, analytical models (GBTM vs. GMM vs. LCGA), and population characteristics precluded formal statistical pooling and limited generalizability. While the overall NOS scores indicated moderate to high quality (range 6–8), the comparability domain, particularly adjustment for key confounders such as baseline depression severity and multimorbidity, was the most frequently rated weak item, whereas attrition handling was often poorly reported, potentially introducing selection bias. This heterogeneity also prevented meaningful assessment of publication bias.

(b) Measurement heterogeneity remains problematic: all studies relied on symptom scales rather than clinical diagnoses, and our post hoc standardization of trajectory labels, while necessary for cross‐study comparison, may not fully align with original study clinical definitions. Specifically, the proportional rescaling approach assumes interval‐level properties and equivalent item content across scales, which may not hold for dementia‐specific (CSDD) or anxiety‐confounded (HADS‐D) instruments. Scales differ in item content (e.g., CSDD emphasizes cognitive‐behavioral symptoms; CES‐D emphasizes affective symptoms), response categories, and cultural applicability. Consequently, our standardized thresholds should be interpreted as heuristic benchmarks for cross‐study comparison rather than clinically validated cutoffs. The use of CES‐D variants (8‐item to 20‐item) may capture distinct symptom dimensions, particularly in populations with high somatic comorbidity or cognitive impairment.

(c) Temporal ambiguity in trajectory modeling, where different studies used varying numbers of follow‐ups (range 3–9) and durations (4–20 years), complicates comparisons of trajectory stability and transition patterns.

(d) The included samples underrepresented low‐income countries and non‐Western populations, and the restriction to Chinese‐ and English‐language publications may have excluded relevant studies in other languages, potentially limiting geographic coverage and generalizability to global aging contexts.

(e) The choice of severity cutoffs, while grounded in established CES‐D thresholds (≥ 16 for clinically significant symptoms and ≥ 24 for severe symptoms), necessarily entails boundary uncertainty at the Low‐to‐Moderate interface. Trajectories with equivalent scores in the 12–16 range could plausibly be classified in either category depending on population norms and clinical context.

(f) A substantial proportion of included studies drew from overlapping cohorts (e.g., CHARLS, ELSA, and HRS). While we treated each publication as an independent observation in our descriptive synthesis, this may overestimate the apparent consistency of trajectory patterns and predictors. Sensitivity analyses restricting to one estimate per cohort yielded consistent median estimates, suggesting that the core findings are not driven by cohort duplication.

## 5 Conclusion

This comprehensive systematic synthesis focused specifically on older adults, extending prior reviews that included all age groups. Although significant methodological heterogeneity precluded quantitative meta‐analyses, the available evidence consistently indicates the existence of several distinct trajectory categories: predominantly stable low‐level trajectories, accompanied by clinically significant chronic high‐symptom and nonlinear subgroups. By identifying modifiable predictors (functional status, social engagement, and lifestyle factors) and non‐modifiable risk factors (genetic susceptibility and childhood adversity), this review provides guidance for developing targeted prevention and intervention strategies. To advance the field, future primary studies should adopt standardized assessment protocols—including consistent use of validated depression scales, core predictor sets, and transparent attrition reporting—and unified analytical methods, thereby enabling precise quantitative meta‐analyses of trajectory prevalence, transition patterns, and their predictors across diverse aging populations.

## Author Contributions

**Yuying Li**: writing – original draft, formal analysis, data curation, conceptualization. **Qiuyan Liu**: Formal analysis, data curation. **Xue Dong**: writing – review and editing, supervision, project administration, funding acquisition.

## Funding

This work was supported by the Project of Scientific Research Program of Education Department of Jilin Province (No. JJKH20250671KJ).

## Ethics Statement

We confirm that ethical approval was not required for this study because it is a systematic review of previously published data. No new data involving human participants were collected for this work.

## Conflicts of Interest

The authors declare no conflicts of interest.

## Data Availability

Data sharing is not applicable to this article as no datasets were generated or analyzed during the current study.
